# Novel Natural Angiotensin Converting Enzyme (ACE)-Inhibitory Peptides Derived from Sea Cucumber-Modified Hydrolysates by Adding Exogenous Proline and a Study of Their Structure–Activity Relationship

**DOI:** 10.3390/md16080271

**Published:** 2018-08-04

**Authors:** Jianpeng Li, Zunying Liu, Yuanhui Zhao, Xiaojie Zhu, Rilei Yu, Shiyuan Dong, Haohao Wu

**Affiliations:** 1 College of Food Science and Engineering, Ocean University of China, Qingdao 266003, China; changjing@stu.ouc.edu.cn (J.L.); liuzunying@ouc.edu.cn (Z.L.); jie86230@163.com (X.Z.); dongshiyuan@ouc.edu.cn (S.D.); wuhaohao@ouc.edu.cn (H.W.); 2 School of Medicine and Pharmacy, Ocean University of China, Qingdao 266003, China; ryu@ouc.edu.cn

**Keywords:** sea cucumber, ACE-inhibitory peptide, molecular docking, structure-activity relationship, plastein reaction

## Abstract

Natural angiotensin converting enzyme (ACE)-inhibitory peptides, which are derived from marine products, are useful as antihypertensive drugs. Nevertheless, the activities of these natural peptides are relatively low, which limits their applications. The aim of this study was to prepare efficient ACE-inhibitory peptides from sea cucumber-modified hydrolysates by adding exogenous proline according to a facile plastein reaction. When 40% proline (*w*/*w*, proline/free amino groups) was added, the modified hydrolysates exhibited higher ACE-inhibitory activity than the original hydrolysates. Among the modified hydrolysates, two novel efficient ACE-inhibitory peptides, which are namely PNVA and PNLG, were purified and identified by a sequential approach combining a sephadex G-15 gel column, reverse-phase high-performance liquid chromatography (RP-HPLC) and matrix-assisted laser desorption/ionization time-of-flight mass spectrometry (MALDI-TOF/MS), before we conducted confirmatory studies with synthetic peptides. The ACE-inhibitory activity assay showed that PNVA and PNLG exhibited lower IC_50_ values of 8.18 ± 0.24 and 13.16 ± 0.39 μM than their corresponding truncated analogs (NVA and NLG), respectively. Molecular docking showed that PNVA and PNLG formed a larger number of hydrogen bonds with ACE than NVA and NLG, while the proline at the N-terminal of peptides can affect the orientation of the binding site of ACE. The method developed in this study may potentially be applied to prepare efficient ACE-inhibitory peptides, which may play a key role in hypertension management.

## 1. Introduction

Hypertension is one of the leading causes of global disease burden [1]. In 2015, hypertension was estimated to affect 874 million adults worldwide (systolic blood pressure ≥ 140 mmHg), causing approximately 4.5 million deaths. Furthermore, the number of patients suffering from hypertension continues to grow [2]. Angiotensin-converting enzyme (ACE) plays a critical role in blood pressure control systems (renin-angiotensin system) as it converts angiotensin I into angiotensin II, leading to the development of hypertension [3,4]. Therefore, it is quite essential to study the inhibition of ACE in order to prevent and manage hypertension.

Currently, chemically synthesized ACE inhibitor drugs, such as captopril, enalapril and lisinopril, are being widely used in clinical applications [5,6,7]. However, pharmaceutical drugs may lead to some side effects, such as changes in taste, coughs and rashes [8,9]. Consequently, it is important to develop new, efficient and safe natural substitutes to reduce the application of chemical drugs. ACE-inhibitory peptides, which are safer than synthetic ACE inhibitors, have been shown to be useful as antihypertensive drugs [10]. Furthermore, they are found in many food protein sources [4,11,12], including sea cucumber (*Acaudina molpadioidea*). The sea cucumber is a particularly good source of bioactive peptides and is rich in proteins that result in high yields of peptides [13,14]. Recently, it has been reported that efficient ACE-inhibitory peptides can be obtained from *Acaudina molpadioidea* protein hydrolysates by a facile plastein reaction [15,16,17]. Plastein reaction was first discovered by Danilevski in 1902 when he added chymotrypsin to protein hydrolysates, which is considered to be a reverse enzymatic reaction. Since then, the plastein reaction has been used widely for many purposes [11], including the modification of some protein hydrolysates to improve their antioxidant properties [18] or ACE-inhibitory activity [19].

A number of structure–activity relationship studies have showed that peptides, such as APP [4], KPLL [20] and VYPFPGPIPNSLPQNIPP [21], exhibit high ACE-inhibitory activity, which suggests the importance of proline in controlling the ACE-inhibitory activity. ACE-inhibitory activity could be increased from 27.8% to 76.4% by adding proline to casein hydrolysates [11]. This effect is probably due to the imidazole ring in proline residues exhibiting a strong affinity for the amino acid residues at the active centers of ACE [22], thus resulting in an improvement in the ACE-inhibitory activity. Therefore, it might be reasonable to assume that adding exogenous proline to the plastein reaction system is an effective method to further enhance ACE-inhibitory activity.

Structural bioinformatics show that the activity of ACE-inhibitory peptides is related to both their molecular masses and amino acid sequences [23]. Some studies showed that short-chain polypeptides with low molecular masses exhibited high ACE-inhibitory activity [24,25]. For example, MVGSAPGVL and LGPLGHQ with small molecular masses exhibited high ACE-inhibitory activity [26]. Intriguingly, some studies indicated that the ACE-inhibitory activity of nonapeptide is significantly higher than that of hexapeptide [27], while the inhibitory activity of hexapeptide is also significantly higher than that of tripeptide [11]. Thus, apart from molecular weight, the amino acid sequence also plays an important role in determining the ACE-inhibitory activity of peptides. Many structure–activity relationship studies indicated the importance of the C-terminal of amino acids in determining the ACE-inhibitory activity. The inhibitory activity of polypeptides is higher when the C-terminal amino acids are aromatic amino acids (Trp, Tyr and Phe) or aliphatic amino acids (Ile, Ala, Leu and Met) [28]. However, there are only a few studies examining the influence of N-terminal amino acids on the ACE-inhibitory activity and their ACE-inhibition mechanism has not been clearly elucidated. Molecular docking simulations can provide further insight into peptide−ACE interactions and provide a deeper understanding of the ACE-inhibition mechanism in peptides.

To the best of our knowledge, the addition of exogenous amino acids to *Acaudina molpadioidea* protein hydrolysates to enhance the activity of natural ACE-inhibitory peptides has not been previously discussed. The aim of this study was to prepare efficient ACE-inhibitory peptides from sea cucumber-modified hydrolysates by adding exogenous amino acids according to a facile plastein reaction. Furthermore, we aimed to identify the ACE-inhibitory peptides with high activities by matrix-assisted laser desorption/ionization time-of-flight mass spectrometry (MALDI-TOF/MS). In addition, molecular docking simulations were conducted to investigate the nature of interactions between the peptides and ACE. This study can provide previously unknown information on the effect of exogenous amino acids on the activity of natural ACE-inhibitory peptides and provide an insight into the peptide structure−ACE inhibition relationship.

## 2. Results and Discussion

### 2.1. Modification of Acaudina molpadioidea Protein Hydrolysates by Adding Exogenous Amino Acids

**Figure 1:**
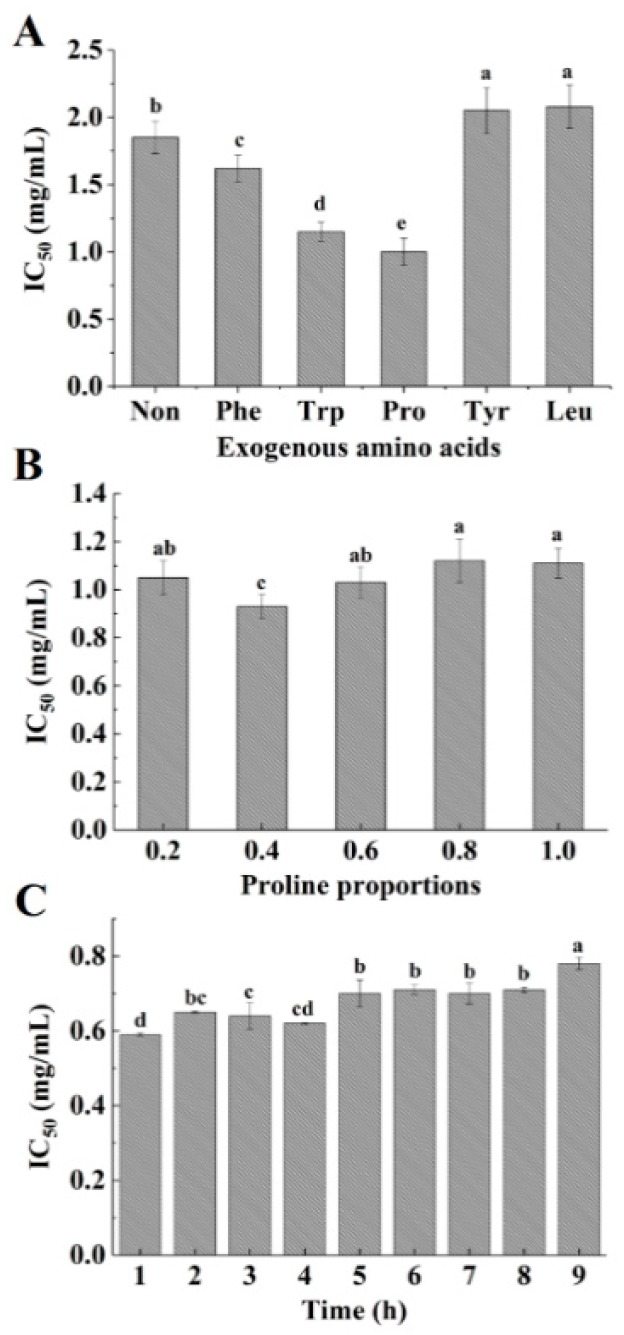
Effect of different amino acids (**A**), proline proportions (**B**) and reaction time (**C**) on the ACE-inhibitory activity. The values of three replicates are shown as mean ± standard deviation. Different lowercase letters indicate significantly different values (*p* < 0.05).

As shown in Figure 1A, the ACE-inhibitory activity of modified hydrolysates significantly increased when compared to original hydrolysates in the presence of exogenous Phe, Trp and Pro at 50% weight proportions to free amino groups. After 6 h of reaction, the maximum ACE-inhibitory activity was found in the Pro group (*p* < 0.05). These results demonstrate that the plastein reaction modified by the addition of exogenous Pro was capable of improving ACE-inhibitory activity. The obtained results are consistent with Sun et al. [11], who reported that the ACE-inhibitory activity of casein hydrolysates was enhanced by adding exogenous Pro. In addition, Bougatef et al. [8] reported that the C-terminal domain of ACE exhibited high hydrophobicity. Hydrophobic amino acids play a crucial role in the inhibition of ACE. The exogenous Tyr, Leu, Phe, Trp and Pro used in this study were hydrophobic amino acids. We deduced that the enhancement in ACE-inhibitory activity upon the addition of Pro groups might be due to the assembly of free Pro in ACE-inhibitory peptides. To confirm this hypothesis, the composition of the free amino acids in the reaction system was investigated. As shown in Table 1, the total free amino acid content constantly decreased with an increase in reaction time, which differs from the case of original hydrolysates. This suggests that free amino acids are involved in the synthesis reaction. The single free amino acid content also exhibited a decreasing trend over a period of 4 h. This is especially true in the case of Pro, whose content decreased to (0.23 ± 0.02) g/(100 mL) after 1 h of reaction (Table 1), suggesting that Pro might be incorporated in the peptide chain during the synthesis reaction. In their study, Sun et al. [11] reported that free amino acids could be introduced into the peptide chain through condensation. From these results, it could be assumed that exogenous Pro was integrated into ACE-inhibitory peptides, which could partly contribute to the high ACE-inhibitory activity.

The plastein reaction is mainly affected by substrate concentration and reaction time [29]. Therefore, the effect of different Pro proportions and reaction time on the ACE-inhibitory activity was determined (Figure 1B,C). When 40% Pro (*w*/*w*, proline/free amino groups) was added, the ACE-inhibitory activity obtained was higher compared to other proportions after 6 h of reaction (IC_50_ = 0.93 ± 0.05 mg/mL) (*p* < 0.05) (Figure 1B). Meanwhile, the ACE-inhibitory activity was the highest (IC_50_ = 0.59 ± 0.03 mg/mL) after 1 h of reaction (Figure 1C). After this time period, the activity decreased with an increase in reaction time. Our results demonstrate that the ACE-inhibitory activity of hydrolysates can be further improved by optimizing the reaction conditions.

### 2.2. Isolation and Purification of Modified ACE-Inhibitory Peptides and the ACE-Inhibitory Activity of Each Fraction

**Figure 2:**
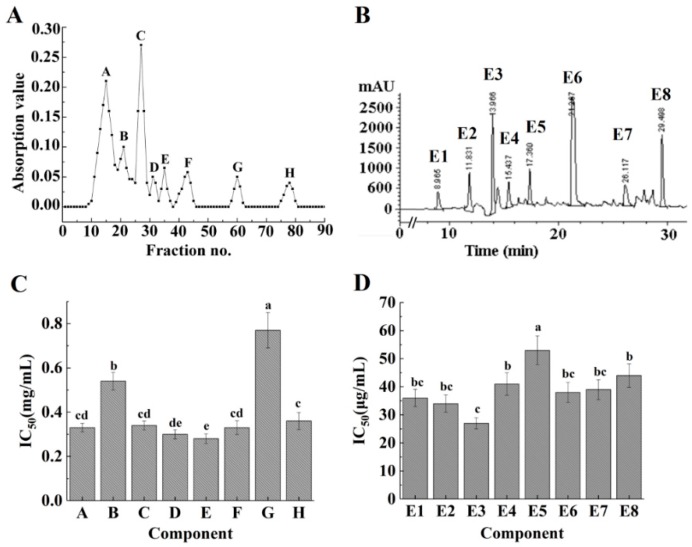
Isolation and purification of modified ACE-inhibitory peptides using a Sephadex G-15 gel column (**A**) and RP-HPLC (**B**). The corresponding ACE-inhibitory activities of each fraction are shown in (**C**,**D**). Different lowercase letters indicate significantly different values (*p* < 0.05).

Chromatographic fractionation is a method that is often used for peptide elution. A Sephadex G-15 gel column was used to fractionate the ACE-inhibitory peptides, before the fractions were pooled to obtain fractions A−H at 220 nm (Figure 2A). After this, these components were collected for determining the ACE-inhibitory activity. Although ACE-inhibitory activity could be observed in all fractions (Figure 2C), fraction E exhibited higher ACE-inhibitory activity compared to others (*p* < 0.05). The fractions with the same molecular mass peaked at the same time in the Sephadex G-15 gel column [30]. Therefore, it was concluded that fraction E may be a mixture and it required further fractionation by RP-HPLC.

RP-HPLC is a frequently used tool for the isolation and purification of polypeptides. Figure 2B summarizes the RP-HPLC analysis results of fraction E in terms of its absorbance at 220 nm. After 8 min of elution, eight major peaks were detected, among which the peak corresponding to fraction E3 exhibited a relatively high intensity. The different fractions showed different activities (Figure 2D) and fraction E3 exhibited higher activity compared to other fractions (IC_50_ = 27 ± 2 µg/mL) (*p* < 0.05). Fraction E3 was purified by 21.85-fold using a two-step purification process (Table 2). This suggests that the ACE-inhibitory activity of *Acaudina molpadioides* peptides can be significantly improved by fractionation and purification.

### 2.3. Identification of the Purified Peptides and Evaluation of Their ACE-Inhibitory Activity

**Figure 3:**
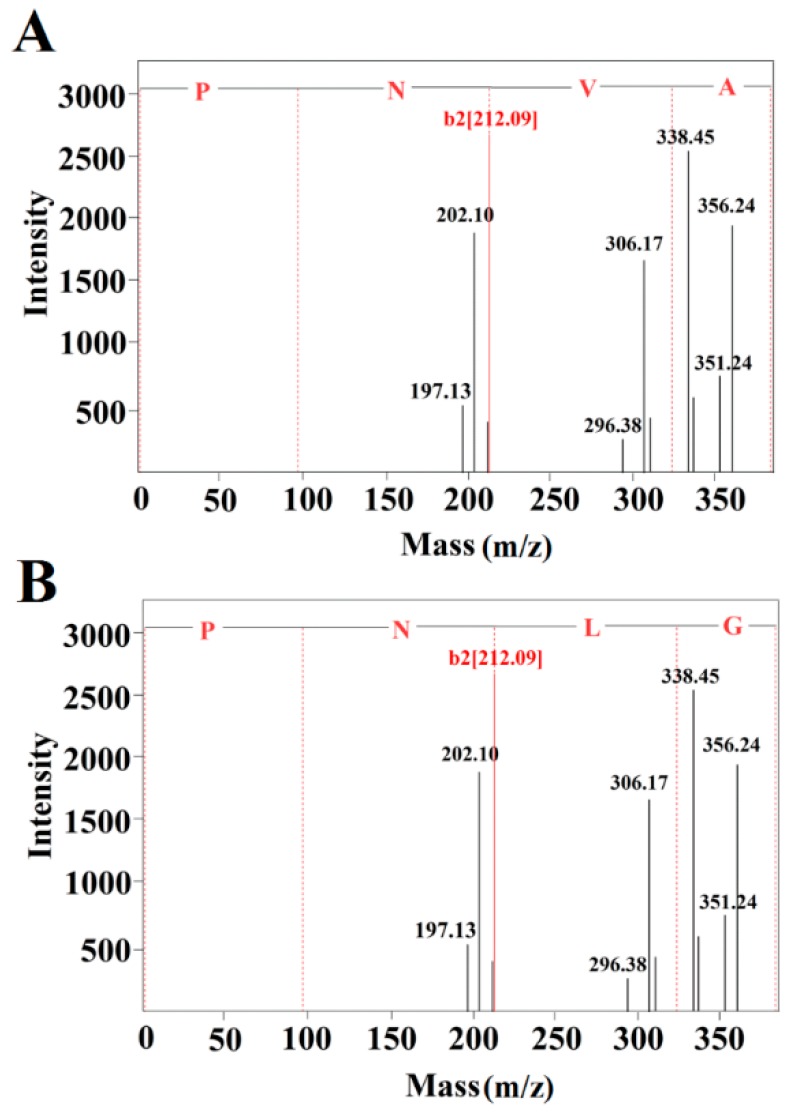
MALDI-TOF/MS spectra of the amino acid sequences of fraction E3. (**A**) peptide PNVA. (**B**) peptide PNLG.

According to the MALDI-TOF analysis of fraction E3, its mass is 400.0966 Da. Furthermore, different peptides were investigated by MALDI-TOF/MS fragmentation analysis. According to the MS spectral data, the peptide sequences were preliminarily matched with those of NVA and NLG, which had been previously characterized in the database. Subsequently, the N-terminal of the peptides was inferred to be Pro by calculating the molecular mass. From these results, we deduced that the whole peptide sequences were PNVA (Figure 3A) and PNLG (Figure 3B), which did not match with the database. Our results indicate that PNVA and PNLG are novel ACE-inhibitory peptides, which are realized in *Acaudina molpadioidea* protein hydrolysates by adding exogenous Pro. These observations are consistent with the results shown in Figure 1 and Table 1.

Subsequently, four peptides, which were namely PNVA, PNLG, NVA and NLG, were synthesized by a chemical method and their ACE-inhibitory activities were determined. As shown in Table 3, the activity of PNVA (IC_50_ = 8.18 ± 0.24 μM) was significantly higher than the activity of PNLG (IC_50_ = 13.16 ± 0.39 μM). Meanwhile, NVA (IC_50_ = 12.69 ± 1.50 μM) exhibited a significantly higher activity than NLG (IC_50_ = 17.45 ± 0.89 μM). Our observations are consistent with those of Jang et al. [28], who reported that the presence of aliphatic amino acids at the penultimate C-terminus, which was namely Val, resulted in significantly increased ACE-binding affinity compared to other amino acids. Moreover, Mizuno et al. [31] reported that the presence of hydrophobic amino acids at the C-terminus positively influenced the ACE-inhibitory activity. The hydrophobic parameters of Ala are significantly higher than those of Gly [32], suggesting that the presence of hydrophobic Ala at the C-terminus exerts more influence on the ACE-inhibitory activity than Gly. This report is consistent with our results, which is shown in Table 3. In addition, Table 3 shows that the ACE-inhibitory activity of PNVA is significantly higher than that of NVA. Similarly, the activity of PNLG is significantly higher than that of NLG. The results indicated that Pro at the N-terminus significantly enhanced the ACE-inhibitory activity. Many structure–activity relationship studies highlighted the influence of C-terminal amino acids on the ACE-inhibitory activity [12,28,31,33]. However, there are very few studies examining the effect of N-terminal amino acids on the ACE-inhibitory activity.

### 2.4. Molecular Docking of ACE-Inhibitory Peptides

To understand the molecular interaction mechanism of the four peptides (PNVA, PNLG, NVA and NLG) against ACE, molecular docking analysis was performed using Molecular Operating Environment (MOE) software. The docking scores of PNVA, PNLG, NVA and NLG were −7.13, −6.77, −5.14 and −5.12 kcal/mol, respectively (Table 4), which suggests that the model can efficiently simulate molecular docking.

**Figure 4:**
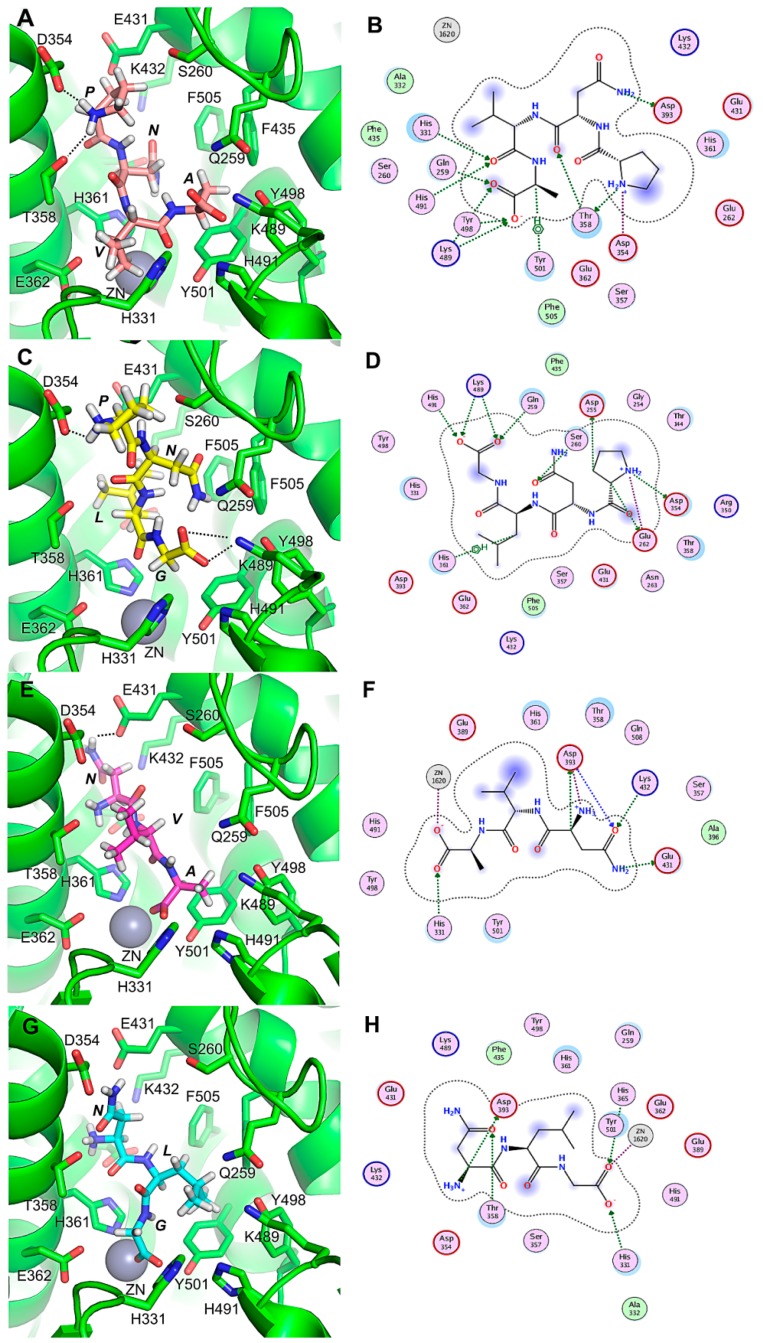
(**A**–**H**) show the binding modes of PNVA, PNLG, NVA and NLG with the ACE, respectively. (**B**,**D**,**F**,**H**) show 2D schematics of the peptide-binding modes. The dashed lines indicate the hydrogen bonds that were formed between the peptide and residues of the binding sites.

The modes of action of ACE-inhibitory peptides include competitive, noncompetitive, uncompetitive and mixed modes. Most ACE-inhibitory peptides act as competitive inhibitors [34]. The binding of ACE to a substrate or competitive inhibitor of amino acid residues is highly specific [11]. Consistent with our experimental studies, the results of molecular docking analysis suggest that the truncation of Pro reduced the binding affinity of PNVA and PNLG as the active peptide PNVA showed the highest binding affinity (Table 4). ACE is a metallo-enzyme with the zinc ion at the active site coordinated by His383, His387 and Glu411 [35]. As shown in Figure 4, PNVA forms eleven hydrogen bonds with the residues Asp354, Thr358, Asp393, His331, His491, Tyr501, Gln259, Tyr498 and Lys489 (Figure 4A,B). Furthermore, PNLG forms ten hydrogen bonds with the residues Asp354, Glu262, Asp255, Ser260, His361, Gln259, Lys489 and His491 (Figure 4C,D). NVA forms seven hydrogen bonds with the residues Glu431, Lys432, Asp391, His331 and Zn1620 (Figure 4E,F), while NLG forms five hydrogen bonds with the residues Asp393, Thr358, His331, His365 and Zn1620 (Figure 4G,H). These results indicate that the ACE active binding sites of different peptides are not identical. This conclusion is consistent with the observations of Liu et al. [35]. Overall, our molecular docking studies indicated that the peptides PNVA and PNLG form more hydrogen bonds with ACE than their corresponding truncated analogs (NVA and NLG), while the four active peptides bind to the catalytic pocket of ACE mainly through a network of hydrogen bonds. The number of hydrogen bonds played an important role in determining the binding affinity of peptides with ACE [17]. For example, lisinopril with higher ACE-inhibitory activity forms nine hydrogen bonds with ACE active residues, which was similar to our results.

**Figure 5:**
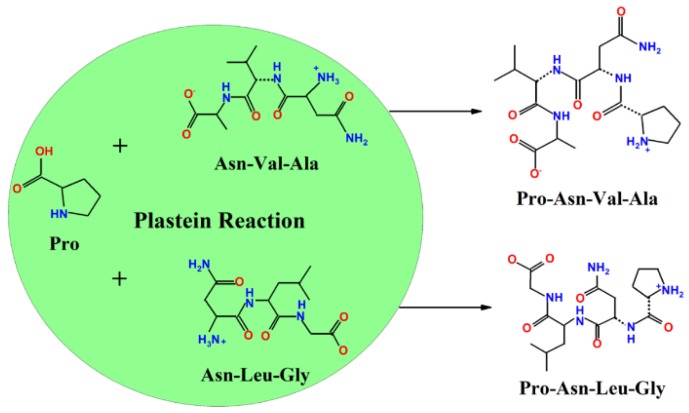
Schematic illustration of the formation of ACE-inhibitory peptides during the plastein reaction.

Intriguingly, the Pro of PNVA forms two hydrogen bonds with Asp354 and Thr358, while the Pro of PNLG forms two hydrogen bonds with Asp354 and Glu262. These results indicate that the Pro at the N-terminal of both PNVA and PNLG plays an essential role in determining their binding affinity. Similarly, Min et al. [36] reported that the interactions of the side chains of Leu and Ile with the hydrophobic residues determined the binding positions of N-terminal residues of LKP and IKP. This subsequently influenced the interaction of the residues of LKP and IKP with the active sites of ACE. In addition, the orientations of the last residues of PNVA and PNLG are different from those of NVA and NLG (Figure 4A,C,E,G). The carboxyl groups of the C-terminals, which are namely Ala and Gly, of PNVA and PNLG are oriented towards Lys489 of the ACE, whereas the carboxyl groups of NVA and NLG are oriented towards Zn^2+^. These results indicate that the Pro at the N-terminal of the peptides could reform the rigid structure of the peptides, further altering the binding sites between peptides and ACE. These are similar to the observations made by Liu et al. [35], who reported that Leu at the N-terminal of LVKF could alter the binding sites of ACE between LVKF and VKF. In summary, our docking studies suggest that Pro at the N-terminal of peptides can affect both the peptide-binding affinity and orientation of the binding site of ACE. The added exogenous Pro was possibly assembled at the N-terminals of their corresponding truncated analogs (NVA and NLG) (Figure 5). Thus, this reforms the rigid structure of the peptides, altering the binding sites and forming a large number of hydrogen bonds with ACE. This subsequently leads to a decrease in the ACE catalytic function.

Numerous studies on hypertensive human volunteers have demonstrated that ACE-inhibitory peptides significantly reduce blood pressure [12]. In addition, some ACE-inhibitory biopeptides have been commercialized [37], which suggests that these biopeptides can be applied for the supplemental treatment of hypertensive patients. In the present study, two novel biopeptides with high ACE-inhibitory activity (PNVA and PNLG) were synthesized by the addition of exogenous Pro. Although the ACE-inhibitory activities of PNVA (IC_50_ = 8.18 ± 0.24 µM) and PNLG (IC_50_ = 13.16 ± 0.39 μM) were much lower than synthetic ACE inhibitor captopril (IC_50_ = 23 nM) [5], the peptides from *A. molpadioidea* were characterized as novel peptides, which are derived from a food source that is eaten daily. Therefore, the ACE-inhibitory peptides (PNVA and PNLG) will be very useful in the preparation of antihypertensive drugs. Meanwhile, their activities were much higher than that of other biopeptides, AGPPGSDGQPGAK (IC_50_ = 420 ± 20 µM) [38], AV (IC_50_ = 956.30 µM) [39] and PPK (IC_50_ > 1000 µM), suggesting the peptides that we prepared have higher ACE-inhibitory activities. The ACE-inhibitory peptides derived from sea cucumber-modified hydrolysates by adding exogenous Pro will play a key role in hypertension management.

## 3. Materials and Methods

### 3.1. Materials and Chemicals

Sea cucumber (*Acaudina molpadioidea*) was purchased from a local market (Qingdao, China) and its water content was determined to be 12.65% per 20 g on average. Hippuryl histidyl leucine (HHL), rabbit-lung ACE and Sephadex G-15 were purchased from Sigma Chemical Ltd (St. Louis, MO, USA). Papain (from papaya) and trypsin (from bovine sources) were purchased from Nanning Pangbo Biological Engineering Ltd (Nanning, China). *O*-Pthaldialdehyde (OPA) was purchased from Beijing Soularbio Technology Ltd. (Beijing, China). Proline (PubChem CID 145742), valine (PubChem CID 6287), tyrosine (PubChem CID 6057), tryptophan (PubChem CID 6305), leucine (PubChem CID 6106) and phenylalanine (PubChem CID 6140) were purchased from Sinopharm Chemical Reagent Ltd. (Shanghai, China). All other chemicals were obtained from local commercial sources and were of the highest purity available.

### 3.2. Acaudina molpadioidea Body Wall Protein Extraction

The protein present in the body wall of *Acaudina molpadioidea* was extracted as described by Jamilah et al. [40]. Briefly, the body wall of *Acaudina molpadioidea* was fully swollen in distilled water over a period of 12 h at 120 °C, before being added to fresh distilled water (10 times in excess) to cut into pieces. During stirring, the protein was extracted at 45 °C over a period of 8 h and centrifuged (10,000× *g*, 20 min) to obtain water-soluble protein solutions. After this, these solutions were freeze-dried using a lyophilizer (Scientz-10nd, Ningbo Scientz Biotechnology Co. Ltd., Ningbo, China).

### 3.3. Acaudina molpadioidea Protein Hydrolysates Preparation

The freeze-dried *Acaudina molpadioidea* body wall protein was initially dissolved in water and its pH was adjusted to 7.0. After this, trypsin (2.5 kU/g protein) and papain (2.5 kU/g protein) were added to this solution and digested in a 50 °C water bath for 4 h. Subsequently, the enzymes were inactivated at 95 °C for 15 min. The suspension was then centrifuged at 8000× *g* and 4 °C for 20 min, after which the supernatant was removed and ultrafiltration precipitation was carried out with a 5 K membrane (Millipore Isopore, Billerica, MA, USA). The obtained precipitate was freeze-dried (Scientz-10nd, Ningbo Scientz Biotechnology Co. Ltd., Ningbo, China) and stored until further use.

### 3.4. Modification of Acaudina molpadioidea Protein Hydrolysates by Plastein Reaction

Different concentrations of exogenous Pro, Phe, Trp, Tyr and Leu (at pre-determined weight ratios of exogenous amino acid to free amino groups) were added to the *Acaudina molpadioidea* protein hydrolysates for the plastein reaction. The reaction conditions were as follows: substrate concentration of 40% (*w*/*v*), temperature of 45 °C and papain dosage of 2.5 kU/g protein. Furthermore, the effect of different proportions and reaction time on the ACE-inhibitory activity was determined.

### 3.5. Changes in the Content of Free Amino Groups during the Plastein Reaction

The OPA method [41], with some modifications, was used to determine the free amino groups in the reaction mixture. The reagent was prepared daily and protected from light. The OPA assay was carried out by adding 3 mL of *Acaudina molpadioidea* hydrolysates to the same volume of OPA reagent (40 mg/mL). After 5 min, the absorbance of the resultant solution was measured at 340 nm using a UV spectrophotometer (UV-2550, Shimadzu, Kyoto, Japan). L-leucine was used as the standard. The equation of the standard curve is Y = 0.3037X + 0.0078, where X is the concentration of free amino acids and Y is the absorption value of the sample.

### 3.6. Determination of the ACE-Inhibitory Activity of Hydrolysates

The ACE-inhibitory activity of the hydrolysates was measured according to a previously reported protocol [14] with slight modifications. Briefly, a sample solution with 40 μL of ACE solution (25 mU/mL) was pre-incubated at 37 °C for 10 min. After this, the mixture was incubated with 50 μL of the substrate (8.3 mM Hip-His-Leu in 50 mM sodium borate buffer containing 0.5 M NaCl at pH of 8.3) for 60 min at the same temperature. The reaction was terminated by the addition of 1.0 M HCl (200 μL). The absorbance of hippuric acid in the incubated solution was determined at 228 nm using a UV-spectrophotometer (UV-2550, Shimadzu, Kyoto, Japan). The IC_50_ value was defined as the concentration of inhibitor that was needed to inhibit 50% of the ACE activity.

### 3.7. Solvent Fractionation of ACE-Inhibitory Peptides by Chromatography

ACE-inhibitory peptides were purified using a Sephadex G-15 column (2.6 cm × 65 cm) and eluted with double distilled water at a flow rate of 1.2 mL/min. The absorbance of the eluent was monitored at 220 nm.

### 3.8. Analysis and Purification of ACE-Inhibitory Peptides in Hydrolysates by RP-HPLC

The ACE-inhibitory activity of peptides in the hydrolysates was analyzed by RP-HPLC on a Zorbax SB-C18 column (9.4 mm × 250 mm, Agilent, Santa Clara, CA, USA) equipped with an Agilent 1260 infinity HPLC system (Agilent Technology, Mississauga, ON, Canada) at a flow rate of 1 mL/min. An acetonitrile gradient from 5% to 30% was used for 40 min to separate different groups of peptides. Chromatographic separation was carried out at 35 °C. The components were collected at the absorbance of 220 nm and their ACE-inhibitory activity was measured.

### 3.9. Mass Spectrometric Analysis and Synthesis of Purified Peptides

The molecular weights of peptides in the filtered hydrolysates were analyzed by MALDI-TOF (TOF 4800, Micromass Company, Manchester, UK). The amino acid sequences were determined in the positive ion mode by de novo sequencing. Subsequently, the peptides were synthesized using the solid phase method and purified by HPLC (ChinaPeptides Co. Ltd., Shanghai, China). The synthesized peptides are listed in Table 3.

### 3.10. Molecular Docking

The initial peptide structures of PNVA, PNLG, NVA and NLG were produced using the xleap module of AMBER16 (University of California, San Francisco, CA, USA) and were subjected to minimization using the Molecular Operating Environment (MOE) software (version 2016) [42]. Molecular docking was performed by MOE using the AMBER10: EHT force field [42]. The crystal structures of ACE (PDB ID: 2XYD) [17] bound with PNVA, PNLG, NVA and NLG were obtained from the Protein Data Bank (http://www.rcsb.org) for the docking studies. The induced-fit docking approach was applied to analyze the side-chain flexibility of residues at the binding sites.

### 3.11. Statistical Analysis

All the data (average of three replicates) are reported in the form of mean ± standard deviation and the results were validated by one-way analysis of variance (ANOVA). Significant differences between the means of the parameters were determined by Duncan’s multiple range tests (*p* < 0.05).

## 4. Conclusions

Upon the addition of exogenous amino acids, it was possible to enhance the ACE-inhibitory activity of *Acaudina molpadioidea*-protein hydrolysates. In particular, *Acaudina molpadioidea*-modified hydrolysates with 40% proline (*w*/*w*, proline/free amino groups) possessed higher ACE-inhibitory activity than the original hydrolysates. Furthermore, the free proline content in the reaction system decreased significantly (*p* < 0.05). In addition, two novel ACE-inhibitory peptides, PNVA and PNLG with IC_50_ values = 8.18 ± 0.24 and 13.16 ± 0.39 μM, were identified for the first time in *Acaudina molpadioidea* protein hydrolysates. Molecular docking showed that PNVA and PNLG form more hydrogen bonds with ACE than NVA and NLG, while the presence of proline at the N-terminals of the peptides can affect the orientation of the binding sites of ACE, leading to a reduction in ACE activity. The exogenous proline is assembled into ACE-inhibitory peptides, with this phenomenon partially contributing to the increase in the ACE-inhibitory activity of natural peptides. In this study, we demonstrated that the addition of exogenous proline to *Acaudina molpadioidea* protein hydrolysates through the plastein reaction is a promising method to enhance the activity of natural ACE-inhibitory peptides. Further studies are necessary to investigate the detailed assembly of exogenous proline using isotopic tracer methods and to prove the in vivo efficacy of ACE-inhibitory peptides in lowering blood pressure. The results of the present study also highlight the potential applications of plastein reaction in preparing antihypertensive drugs.

## Figures and Tables

**Table 1:** The variations of free amino acid groups during plastein reaction.

Amino Acids	Original	1 h g/(100 mL)	4 h g/(100 mL)
Thr	0.05 ± 0.02	0.04 ± 0.01	0.03 ± 0.01
Ser	0.01 ± 0.01	0.01 ± 0.01	0.01 ± 0.01
Glu	0.03 ± 0.01	0.02 ± 0.01	0.02 ± 0.01
Gly	0.05 ± 0.02	0.04 ± 0.02	0.04 ± 0.02
Ala	0.04 ± 0.02	0.03 ± 0.01	0.03 ± 0.01
Cys	0.06 ± 0.02	0.05 ± 0.02	0.05 ± 0.01
Val	0.03 ± 0.01	0.03 ± 0.01	0.02 ± 0.01
Met	0.01 ± 0.01	0.01 ± 0.01	0.01 ± 0.01
Ile	0.02 ± 0.01	0.01 ± 0.02	0.01 ± 0.01
Leu	0.05 ± 0.02	0.04 ± 0.01	0.04 ± 0.02
Tyr	0.03 ± 0.01	0.02 ± 0.01	0.02 ± 0.01
Phe	0.03 ± 0.01	0.02 ± 0.01	0.02 ± 0.01
Lys	0.02 ± 0.01	0.01 ± 0.01	0.02 ± 0.01
His	0.01 ± 0.01	0.01 ± 0.01	0.01 ± 0.01
Arg	0.14 ± 0.03	0.11 ± 0.02	0.11 ± 0.02
Pro	0.29 ± 0.04 ^a^	0.23 ± 0.02 ^b^	0.2 ± 0.02 ^b^
Total	0.90 ± 0.09 ^a^	0.69 ± 0.08 ^b^	0.66 ± 0.08 ^b^

Mean ± SD (*n* = 3). Values with different superscript letters are significantly different (*p* < 0.05).

**Table 2:** Purification procedure of ACE-inhibitory peptides.

Component	Purification	IC_50_ (mg/mL)	Purification Fold
Hydrolysates	Ultrafiltration	0.590 ± 0.030 ^a^	1.00
E	Sephadex G-15	0.288 ± 0.013 ^b^	2.05
E3	RP-HPLC	0.027 ± 0.002 ^c^	21.85

Mean ± SD (*n* = 3). Values with different superscript letters are significantly different (*p* < 0.05).

**Table 3:** IC_50_ values and amino acid sequences of peptides, based on the algorithm for peptide sequencing de novo.

Sequence	Molecular Mass (Da)	ACE IC_50_ (µM) ^2^
PNVA ^1^	399.45	8.18 ± 0.24 ^a^
PNLG ^1^	399.45	13.16 ± 0.39 ^b^
NVA	302.33	12.69 ± 1.50 ^b^
NLG	302.33	17.45 ± 0.89 ^c^

^1^ peptides from *Acaudina molpadioidea* protein hydrolysates. ^2^ Mean ± SD (*n* = 3). Values with different superscript letters are significantly different (*p* < 0.05).

**Table 4:** Docking score and experimental binding affinity of peptides.

Peptide	Docking Score (kcal/mol)	Experimental Binding Affinity (kcal/mol) ^1^
PNVA	−7.13	−2.34
PNLG	−6.77	−2.47
NVA	−5.14	−2.46
NLG	−5.12	−2.54

^1^ Ligand binding affinities were calculated using the equation: ΔG = −RT ln IC_50_, where R = 8.314 J·mol^−1^·K^−1^ and T = 300 K.
